# Road to entire insulation for resonances from a forced mechanical system

**DOI:** 10.1038/s41598-022-25691-4

**Published:** 2022-12-07

**Authors:** Guangnan Zhu, Qingjie Cao, Zhenkun Wang, Yuntian Zhang, Yushu Chen, Ko-Choong Woo

**Affiliations:** 1 School of Astronautics, Harbin Institute of Technology, Harbin, 150001 China; 2 No. 703 Research Institute of CSSC, Harbin, 150078 China; 3 The University of Nottingham Malaysia Campus, Semenyih, 43500 Malaysia

**Keywords:** Acoustics, Mechanical engineering

## Abstract

The effective solution to avoid machinery damage caused by resonance has been perplexing the field of engineering as a core research direction since the resonance phenomenon was discovered by Euler in 1750. Numerous attempts have been performed to reduce the influence of resonance since the earlier of last century, by introducing a nonlinear structure or a closed-loop control system. However, the existed methodologies cannot eliminate resonance completely even extra problems were introduced inevitably, which means the technical choke-point of resonance-free remains unsolved. Here we propose a designable archetype model, which establishes a mapping between the mechanical properties and its structure. A general inverse method for structure construction is proposed based upon the required property for the system with quasi-zero stiffness of any designed finite order and the zero-stiffness properties. It is shown that an ellipse trajectory tracking of the designed model is the sufficient and necessary condition to satisfy the zero-stiffness property. Theoretical analysis shows that no resonant response happens in a zero-stiffness system to the full-band frequency excitation, or equivalently, the system can completely isolate the energy transfer between the load and environment, when the damping ratio approaches zero. Finally, an experimental rig for the prototype structure is built up according to the sufficient and necessary condition of the zero-stiffness system, for which the special dynamic behaviours are verified through experiments of frequency-sweep and random-vibration as well. Experimental results show that the prototype of the initial vibration isolation frequency of zero-stiffness system is much lower than 0.37 Hz, and the vibration attenuation of the proposed model is about 16.86 dB, 45.63 dB, and 112.37 dB at frequencies of 0.37 Hz, 1 Hz, and 10 Hz, respectively. The distinguished geometric structure of the zero-stiffness system leads to a new inspiration for the design of resonance-free in metamaterial unit and the inverse method can even adapt the design for a more targeted applications based on an arbitrary complex dynamic requirement.

Vibrations are ubiquitous. In many application scenarios, people use vibration to bring great convenience to our life. For instance in providing power in sensors^1^, the piezoelectric energy harvesters^2^, and in inductively coupled electromechanical systems^3^. However, the ability of a supporting system to eliminate the damage caused by the resonance discovered by Leonhard Euler in 1750^4^, is also of great importance^5,6^, especially in the fields of anti-seismic of building^7,8^, for the destruction energy generated by the earthquake is mainly concentrated in the low-frequency band below 10 Hz, while there are often many low-frequency vibration modals exist in large buildings^9^; the fields of modal test of large flexible structures^10,11^, for the large flexible structures often have the characteristics of large mass and low-frequency simultaneously, which poses a problem to the supporting structure that it cannot provide sufficient bearing capacity if its stiffness is low, or the accuracy of modal testing will be affected if its stiffness is high^12^; the fields of the ground simulation of zero- or micro-gravity environment^13–15^, for the stiffness from any gravity compensation scheme will introduce an error into the simulation, which means that it is necessary to propose a gravity compensation with zero-stiffness characteristics^16^; the fields of the low- or ultra-low-frequency vibration isolation^17–19^, for the traditional vibration isolation scheme always expresses vibration amplification phenomenon caused by resonance in the frequency band lower than its initial vibration isolation frequency; and etc. Plenty of innovative researches have been carried out to understand and develop the means to decouple resonance of supporting structures from systems, mainly through the methods of introducing high damping, flexible supporting, closed-loop control system, or nonlinear structure. Snyder^20^ studied the performance of magnetorheological damper under external excitation; Deringo^21^ studied the seismic isolation ability of the rubber with high damping for the buildings. David^22^ proposed a supporting structure with near-zero stiffness characteristic to simulate the unconstrained (free-free) boundary conditions for the ground vibration testing of spacecraft. Olsson^23^ studied a vibration isolator based upon a feedback control system for automotive engine; Yuan^24^ proposed a general dynamics and control model for active vibration control. Molyneux^25^ first proposed the quasi-zero stiffness system in 1957 based on the principle of parallel connection of inclined spring and vertical spring; Xu^26^ designed a quasi-zero stiffness isolator based on magnet connecting rod. Despite these advances, mechanical resonances remain in engineering systems irremovable. Reduction of resonance amplitude by increasing damping usually compromises the isolation in high-frequencies^27^. Unaccepted, large deformations might occur in a flexible supporting system with a heavy load^28^. The complex structure of active control technology causes some defects such as high-energy consumption, high cost, and time delay of signal transmission^29–31^, which make it unable to be widely used in some non-sophisticated fields. The resonance-frequency moves towards left (lower-frequency) in a nonlinear system with the characteristic of High-Static and Low-Dynamic (HSLD) stiffness or Quasi-Zero Stiffness (QZS); however, a severer resonance may occur in some degree than it in a linear system^32^. On the other hand, zero-stiffness structures exist, but are unsuitable for implementing into mechanical systems to remove resonance^33^, since they are either designed for small loads, for the microactuators^34^, instance the Anglepoise Lamp^35^; or no loads at all, for instance the twisting rod^36^ and the bistable cylindrical shell^37^.

Thus, a specific kind of device with the following features would pave the way towards ultra-stable engineering of mechanical systems:

1. With vibration attenuation in full-band frequency;
2. With large bearing capacity;
3. Without any energy input;
4. Without resonance phenomena.

Here, we demonstrate an inverse construction method to enable a system to achieve the specific function based upon the dynamic characteristic requirement in an arbitrary application scenario, through the designing of the trajectory *S* of the designable archetype shown in Fig. 1a. The passive archetype represents the potential to derive the strict zero-stiffness (ZS) characteristics, which indicates unprecedented immunity to disturbance in full-band frequency. This is the first time that such a system has been demonstrated, and we will present both the theoretical framework and experimental demonstration (Supplementary Videos 1, 2).

## Archetypal model

**Figure 1:**
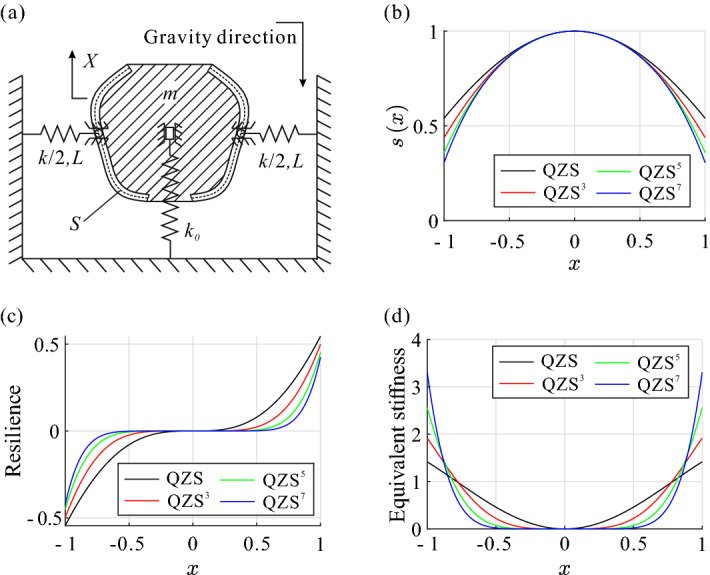
Schematic of the archetypal model and the characteristics under the different order QZS conditions (**a**) for the schematic diagram of the designable model, (**b**) for the dimensionless trajectory curves $s\left( x\right)$ to construct the QZS, QZS$^3$, QZS$^5$, and QZS$^7$, which are plotted in black, red, green, and blue, respectively, and the following are the same, (**c**) for the dimensionless resilience curves of the different order QZS systems, (**d**) for the dimensionless equivalent stiffness curves of the different order QZS systems.

The archetype shown in Fig. 1a comprises a mass block, a vertical supporting spring, and a pair of horizontal springs. The movement of the mass block is restricted to the vertical direction, and all the mass, *m*, is lumped at the geometric centre. The deformation of each the horizontal spring, with the stiffness *k*/2 and the equilibrium length *L*, is restricted to the horizontal direction. The vertical spring connects the basement and the geometric centre of the block, with the stiffness $k_0$. Each of the horizontal spring connects the basement and a slider, which is installed in a frictionless slideway with a bilateral symmetrical shape (determined by the trajectory function $S\left( X\right) , X\in \left( -l_0,l_0\right)$, denoted as the dotted curve). It is easy to prove that the dimensionless dynamic equation of the designable archetype can be written as

1 $$\begin{aligned} \ddot{x}+\kappa \left( s\left( x\right) -b\right) s'\left( x\right) +x+\eta =0, \end{aligned}$$

by letting $x=\frac{X}{l_0}\in \left( -1,1\right)$, $b=\frac{B}{l_0}\in {\mathbb {R}}$, $\omega _0^2=\frac{k_0}{m}$, $\omega _1^2=\frac{k}{m}$, $\tau =\omega _0 t\in \left[ 0,+\infty \right)$, $b_0=\frac{B_0}{l_0}$, $\eta =\frac{g}{\omega _0^2l_0}-b_0$, $\kappa =\frac{\omega _1^2}{\omega _0^2}\in \left[ 0,+\infty \right)$, and $s\left( x\right) =\frac{1}{l_0}S\left( l_0x\right)$, in which *B* is the horizontal distance between the geometric centre of the mass block and the free end of one of the horizontal spring at its relaxed state; $B_0$ expresses the compression value of vertical spring in initial state; $g\approx \, 9.81$ m/s$^{2}$ is the gravitational acceleration; ${\dot{x}}$ and $\ddot{x}$ represent the first and the second order derivative of the dimensionless displacement *x* to the dimensionless time $\tau$, respectively; and $s'\left( x\right)$ represent the first order derivative of the dimensionless trajectory function $s\left( x\right)$ to the dimensionless displacement *x*, and the following are the same.

## Finite-order QZS

The definition of the *n*-order quasi-zero stiffness system is proposed by analogy with the definition of the QZS system, as following

### Definition 1

A system is called the *n*-order ($n=2i-1$, $i\in {\mathbb {N}}^+$) quasi-zero stiffness system, which can be recorded as QZS$^n$ and the upper right label can be omitted when $n=1$, if and only if it satisfies the following conditions simultaneously

1. The resultant force $f\left( x\right)$ is at least $\left( n+2\right)$-order continuous differentiable;
2. The resultant force satisfies the condition of
  2 $$\begin{aligned} {\left\{ \begin{array}{ll} T_{i}=0,i<n+2,i\in {\mathbb {N}}^+, \\ T_{n+2}\ne 0,n\ge 1, \end{array}\right. } \end{aligned}$$
  where $T_i$ is the coefficient of the term $x^i$ in Taylor’s expansion of the function $f\left( x\right)$, in a neighborhood of $x=0$.

The solution of QZS$^n$ ($n\in {\mathbb {N}}^+$) conditions for system (1) can be studied by assuming the trajectory satisfies

3 $$\begin{aligned} {_ns}\left( x\right) =\sum _{\varepsilon =1}^{\tfrac{n+3}{2}}\left( a_\varepsilon \cos \varepsilon x+b_\varepsilon \sin \varepsilon x\right) , \end{aligned}$$

in which $\left\{ a_\varepsilon \right\}$ and $\left\{ b_\varepsilon \right\}$ are undetermined coefficients. It is easy to prove that the first condition of Definition1 is satisfied, based upon the infinitely continuous differentiability of ${_ns}\left( x\right)$.

Defining two groups of parameters $s_q$ and $_ns_q$ ($q\in {\mathbb {N}}$) represent the value of $\frac{{\mathrm{d}}^qs\left( x\right) }{{\mathrm{d}}x^q}\big |_{x=0}$ and $\frac{{\mathrm{d}}^q{_ns}\left( x\right) }{{\mathrm{d}}x^q}\big |_{x=0}$, respectively, to analyse the second condition of Definition1. The series $s_q$ can be determined by parameters $\eta$, *b*, $\kappa$, and $s_0$ through a recursive relation as following

4 $$\begin{aligned} {\left\{ \begin{array}{ll} s_1=\frac{\eta }{\kappa \left( b-s_0\right) },s_2=\frac{\kappa s_1^2+1}{\kappa \left( b-s_0\right) }, \\ s_{i+1}=\frac{1}{b-s_0}\sum \limits _{j=1}^{i}C_i^js_{i-j+1}s_{j}, 2\le i\le n+1, \end{array}\right. } \end{aligned}$$

based upon the first equation of (2). Apparently, the second equation of (2) always holds by letting

5 $$\begin{aligned} _ns_i=s_i, \end{aligned}$$

and the following vector

6 $$\begin{aligned} {\textbf{C}}= \begin{bmatrix} a_1&a_2&\cdots&a_{\tfrac{n+3}{2}}&b_1&b_2&\cdots&b_{\tfrac{n+3}{2}} \end{bmatrix}^{\mathrm{T}}, \end{aligned}$$

consisted of the undetermined coefficients, always has a solution written as

7 $$\begin{aligned} {\textbf{C}}={\textbf{V}}^{-1}\cdot {\textbf{S}}, \end{aligned}$$

to determine a dimensionless trajectory function adapt for QZS$^n$ system, in which

8 $$\begin{aligned}{} & {} {\textbf{S}}= \begin{bmatrix} s_0&\quad s_2&\quad \cdots&\quad s_{n+1}&\quad s_1&\quad s_3&\quad \cdots&\quad s_{n+2} \end{bmatrix}^{\mathrm{T}}, \end{aligned}$$

9 $$\begin{aligned}{} & {} {\textbf{V}}= \begin{bmatrix} {\mathbf {V}}_{\mathbf{A}} &{}\quad {\textbf{0}} \\ {\textbf{0}} &{}\quad {\mathbf {V}}_{\mathbf{B}} \end{bmatrix}, \end{aligned}$$

and the elements located at the row *i* and the column *j* in the matrices ${\mathbf {V}}_{\mathbf{A}}$ and ${\mathbf {V}}_{\mathbf{B}}$ with $\frac{n+3}{2}$ rows and columns are expressed as $\left( -j^2\right) ^{i-1}$ and $j\left( -j^2\right) ^{i-1}$, respectively.

The dimensionless trajectory function $s\left( x\right)$ of the QZS, QZS$^3$, QZS$^5$, and QZS$^7$ systems are shown in the Fig. 1b as the black, red, green, and blue curves, respectively, and the force and stiffness curves of the corresponding systems are plotted in the same colours, shown in Fig. 1c,d, respectively. It is clearly that the flat interval, where the force or stiffness closes to zero, broadens as the order of QZS increases. Additionally, does it is possible to enlarge this interval up to the whole domain and to flat the curve along the axis strictly? The answer is yes, as illustrated in the following section.

## Infinite-order QZS

### Inverse construction method

The inverse problem of the previous section reflect a strong research significance, that a general method can be applied to construct mechanical structures by designing of a curve $s\left( x\right)$, based on arbitrary resilience functions. The solution can be acquired as following

10 $$\begin{aligned} \kappa \left( \left( s\left( x\right) -b\right) ^2-\left( s_0-b\right) ^2\right) =2\int _0^x\left( f\left( t\right) -\eta \right) {\mathrm{d}}t-x^2, \end{aligned}$$

by considering the initial value problem of the differential equations as following

11 $$\begin{aligned} {\left\{ \begin{array}{ll} \kappa \left( s\left( x\right) -b\right) s'\left( x\right) +x+\eta =f\left( x\right) , \\ s\left( 0\right) =s_0,x\in \left( -1,1\right) . \end{array}\right. } \end{aligned}$$

### ZS system

Consider a QZS$^n$ system satisfies the Definition 1 based upon (1), and the equation of the dimensionless trajectory can be acquired as

12 $$\begin{aligned} \frac{\left( x+\eta \right) ^2}{\kappa r^2}+\frac{\left( y-b\right) ^2}{r^2}=1+\frac{2}{\kappa r^2}\sum \limits _{i\ge n+2}\frac{T_i}{i+1}x^{i+1}, \end{aligned}$$

where $$y=s\left( x\right) $$ and $$r^2=\frac{\eta ^2}{\kappa }+\left( s_0-b\right) ^2$$.

It is obviously that the necessary and sufficient condition of the infinity-order QZS (QZS$^\infty$) of the system (1) can be expressed as that the dimensionless trajectory satisfied the equation as following

13 $$\begin{aligned} \frac{\left( x+\eta \right) ^2}{\kappa r^2}+\frac{\left( y-b\right) ^2}{r^2}=1, \end{aligned}$$

by proving the following proposition.

#### Proposition 1

*The necessary and sufficient condition for ZS* (*or QZS*$^\infty$) *of the system* (1) *can be expressed as that the trajectory function* $y=s\left( x\right)$ *satisfied the equation* (13).

#### Proof

Consider a QZS$^n$ system satisfy the Definition1 based upon (1), in which the function of resultant force $f\left( x\right)$ can be written as following

14 $$\begin{aligned} f\left( x\right) =\sum \limits _{i\ge n+2}T_ix^i, \end{aligned}$$

which satisfied $n=2j-1 \left( j\in {\mathbb {N}}^{+}\right)$ and

15 $$\begin{aligned} T_i=\frac{f_i}{i!}, \end{aligned}$$

where $f_i$ ($i\in {\mathbb {N}}$) represent the value of $\frac{{\mathrm{d}}^if\left( x\right) }{{\mathrm{d}}x^i}\bigg |_{x=0}$, and the following are the same. Substituting (14) into (10), the equation of the trajectory can be acquired as (12).

It is easy to prove that the coefficient $c_i$ always exists based upon the infinite differentiability of $f\left( x\right)$, and satisfies the relation as following

16 $$\begin{aligned} c_i=\frac{T_i}{i+1}x^{i+1}. \end{aligned}$$

Compare these two coefficients $c_i$ and $c_{i+1}$ by

17 $$\begin{aligned} \frac{c_{i+1}}{c_i}=\frac{\frac{T_{i+1}}{i+2}x^{i+2}}{\frac{T_i}{i+1}x^{i+1}}. \end{aligned}$$

Substituting (15) into (17), there is

18 $$\begin{aligned} \frac{c_{i+1}}{c_i} =\frac{\frac{f_{i+1}}{\left( i+2\right) !}x^{i+2}}{\frac{f_i}{\left( i+1\right) !}x^{i+1}} =\frac{f_{i+1}}{f_i}\frac{x}{\left( i+2\right) }. \end{aligned}$$

Because the conditions of $x\in \left( -1,1\right)$ and $f_i\ne 0$ based on the Definition 1, it is easy to prove that $\frac{f_{i+1}}{f_i}x$ is bounded. Then for the QZS$^\infty$, the limit

19 $$\begin{aligned} \lim \limits _{i\rightarrow \infty }\frac{c_{i+1}}{c_i} =\lim \limits _{i\rightarrow \infty }\frac{f_{i+1}}{f_i}\frac{x}{\left( i+2\right) } \end{aligned}$$

exists and equals to 0, which means

20 $$\begin{aligned} l_{n+1}=\lim \limits _{n\rightarrow \infty }c_{n+1}={\mathrm{o}}\left( \lim \limits _{n\rightarrow \infty }c_n\right) ={\mathrm{o}}\left( l_n\right) , \end{aligned}$$

where ${\mathrm{o}}\left( l_n\right)$ represents the higher order infinitesimal of $l_n$. Thus, the following equation must be hold

21 $$\begin{aligned}&\lim \limits _{n\rightarrow \infty }\sum \limits _{i\ge n+2}\frac{T_i}{i+1}x^{i+1}=\sum \limits _{i\ge n+2}\lim \limits _{n\rightarrow \infty }\frac{T_i}{i+1}x^{i+1}\\&\quad =\sum \limits _{i\ge n+2}\lim \limits _{n\rightarrow \infty }c_i=\sum \limits _{i\ge n+2}l_i\\&\quad =l_{n+2}+{\mathrm{ o}}\left( l_{n+2}\right) +\cdots =l_{n+2}\\&\quad =\lim \limits _{n\rightarrow \infty }T_{n+2}\frac{x^{n+3}}{n+3}=0. \end{aligned}$$

Substitute (21) into the equation (12), then the equation (13) can be obtained, and the necessity of the condition in the proposition is proved.

Substituting (13) and $r^2=\dfrac{\eta ^2}{\kappa }+\left( s_0-b\right) ^2$ into (10) and simplifying it, we can get

22 $$\begin{aligned} \int _0^x\left( f\left( t\right) -\eta \right) {\mathrm{d}}t=-\eta x. \end{aligned}$$

It is obviously that the function $f\left( t\right)$ must satisfy

23 $$\begin{aligned} f\left( t\right) \equiv 0, \end{aligned}$$

based upon the solution of the equation (22), which indicates that the sufficiency of the condition in the proposition is proved. $\square$

There is an interesting conclusion based on the equation (13), that is, if a system approaches to QZS$^\infty$, the curve $y=s\left( x\right)$ will approach to a circle (when $\kappa =1$), or an ellipse with the semi-major (or semi-minor) and the semi-minor (or semi-major) axes equal to $2\sqrt{\kappa }\left| r\right|$ and $2\left| r\right|$, when $\kappa >1$ (or $\kappa <1$), respectively. The special ZS characteristic of this kind of system means that the resilience of the system is not related to the displacement. Equivalently, this kind of system may provide a suspension state to the loading, for its characteristic without any resistance to elastic deformation. The definition of the ZS system is given as following.

#### Definition 2

A system is called the ZS (or QZS$^\infty$) system, if and only if it satisfies the following condition

24 $$\begin{aligned} k\left( x\right) =\frac{{\mathrm{d}}f\left( x\right) }{{\mathrm{d}}x}\equiv 0,x\in \left( -1,1\right) , \end{aligned}$$

### Dynamics characteristics

Considering the system with a pair of elliptical trajectories as following

25 $$\begin{aligned} \ddot{x}+\left( 1-\alpha +\frac{\beta }{\sqrt{1-x^2}}\right) x=0, \end{aligned}$$

with the equilibria satisfying

26 $$\begin{aligned} {\left\{ \begin{array}{ll} x_0=0, \\ x_\pm =\pm \sqrt{1-\left( \frac{\beta }{\alpha -1}\right) ^2}, 0<\frac{\beta }{\alpha -1}\le 1, \end{array}\right. } \end{aligned}$$

where $a=\frac{R_y}{R_x}\in \left( 0,+\infty \right)$, $\alpha =\kappa a^2\in \left[ 0,+\infty \right)$, $\beta =\kappa ab\in {\mathbb {R}}$, and $R_x$ and $R_y$ express the axes of the ellipse in *x* and *y* directions, respectively.

**Figure 2:**
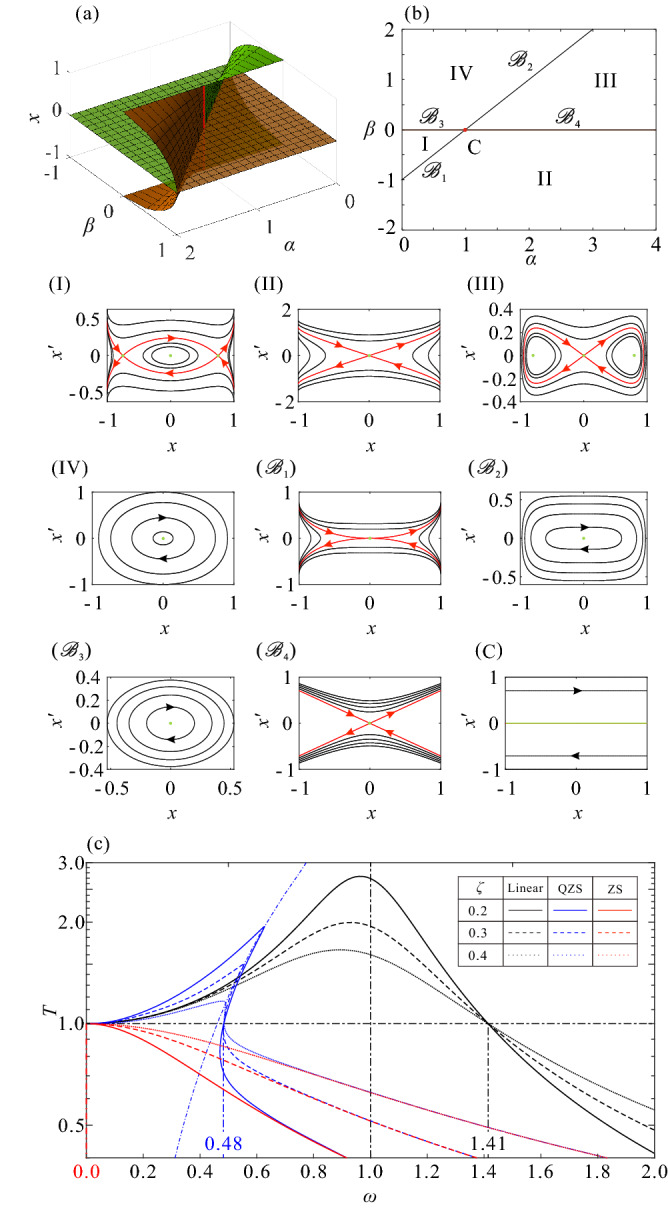
Dynamics characteristics of the ZS and its disturbed systems (**a**) for the equilibrium surface in space $\left( \alpha ,\beta ,x\right)$, (**b**) for the transition sets divide parameter $\left( \alpha , \beta \right)$ plane into four persistent regions, marked I, II, III, and IV, for which the corresponding phase portraits I, II, III, and IV for persistent, ${\mathscr {B}}_1$, ${\mathscr {B}}_2$, ${\mathscr {B}}_3$, and ${\mathscr {B}}_4$ for nonpersistent, while ${\mathrm{C}}$ for catastrophe point, (**c**) for the force transmissibility curves of linear, QZS, and ZS systems with different damping ratios $\zeta$.

The equilibria of the system (25) are plotted in Fig. 2a, in which the surfaces in orange and green separately show the stable and unstable equilibria. The transition sets consisted by bifurcation values can be acquired in the parameter space $\left( \alpha ,\beta \right)$, shown in Fig. 2b, in which there are two lines $L_1:{\mathscr {B}}_1\cup {\mathscr {B}}_2$ and $L_2:{\mathscr {B}}_3\cup {\mathscr {B}}_4$, on which the system is structurally unstable. The two lines divide the parameter space into four distinct regions marked by I, II, III, and IV, respectively, in which the system is structurally stable. The transition sets bifurcate at the catastrophe point ${\mathrm{C}}:\left( 1,0\right)$ into four branches of a bifurcation, which are caused by linear degradation marked as ${\mathscr {B}}_3$ and ${\mathscr {B}}_4$, a subcritical and a supercritical pitchfork bifurcation marked as ${\mathscr {B}}_1$ and ${\mathscr {B}}_2$, respectively. The phase portraits for parameters taken all over the spaces are plotted in Fig. 2 using the Hamiltonian function, marked the same as on the corresponding parameter set in the transition sets, Fig. 2b. The green dots are the centres and saddles, which connecting the corresponding heteroclinic and homoclinic orbits are marked with red.

Especially, when the parameters’ combination $\left( \alpha , \beta \right)$ falls into the catastrophe point ${\mathrm{C}}$, the equilibria of the system are indicated as the red line in Fig. 2a and the corresponding phase portrait is shown in Fig. 2c, which exhibits an infinite number of equilibria. This means that the potential energy stored in the system is always constant, implying that it cannot transfer any energy as a medium. In this case, the loading is completely isolated from the external, without any energy transfer, and the system satisfy the ZS system definition of Definition 2.

### Effect of damping ratio

The transmissibility curves of the linear^38^, QZS, and ZS systems separately determined by the following equations

27 $$\begin{aligned} T_{\mathrm{linear}}= & {} \sqrt{\frac{1+\left( 2\zeta \omega \right) ^2}{\left( 1-\omega ^2\right) ^2+\left( 2\zeta \omega \right) ^2}}, \end{aligned}$$

28 $$\begin{aligned} T_{{\mathrm{QZS}}}^2= & {} \frac{\left( 1-\alpha +\frac{\beta }{\pi }Z\left( \dfrac{Tf}{\omega ^2}\right) \right) ^2+\left( 2\zeta \omega \right) ^2}{\left( 1-\alpha +\frac{\beta }{\pi }Z\left( \dfrac{Tf}{\omega ^2}\right) -\omega ^2\right) ^2+\left( 2\zeta \omega \right) ^2}, \end{aligned}$$

and

29 $$\begin{aligned} T_{\mathrm{ZS}}=\frac{2\zeta }{\sqrt{\omega ^2+4\zeta ^2}}, \end{aligned}$$

which are shown in Fig. 2c marked in black, blue, and red, respectively, and the solid, dashed, and dotted curves separately show the system with damping ratio satisfied $\zeta =0.2$, 0.3, and 0.4. Evidently, the QZS system can produce vibration attenuation effect at least above $\omega _Q\approx 0.48$, while the ZS system can reduce the initial vibration isolation frequency to $\omega _Z=0$. It is widely known that the increase in the damping ratio will improve the low-frequency isolation capability of the system by suppressing the resonance phenomenon, at the expense of high-frequency isolation performance. And the demarcation point between the high- and the low-frequency section of a system can be considered as the initial vibration isolation frequency, deservedly. Hence, the global isolation performance of ZS system will only increase with the decrease in the damping ratio $\zeta$, for its characteristic of $\omega _Z=0$ which indicates that the ZS system does not have a low-frequency band and resonance phenomenon. Further, the transmissibility of the ZS system can be expresses as a constant function as following

30 $$\begin{aligned} T_{{\mathrm{ZS}}}\equiv 0, \end{aligned}$$

by substituting $\zeta =0$ into the (29) and simplify. It is means that the system will not have any ability of energy transmission, and will outright eliminate the mechanical resonance by completely isolating the energy transfer between the load and environment, to realize the immune effect of vibration in full frequency band.

## Experimental investigations

### Introduction of prototype

**Figure 3:**
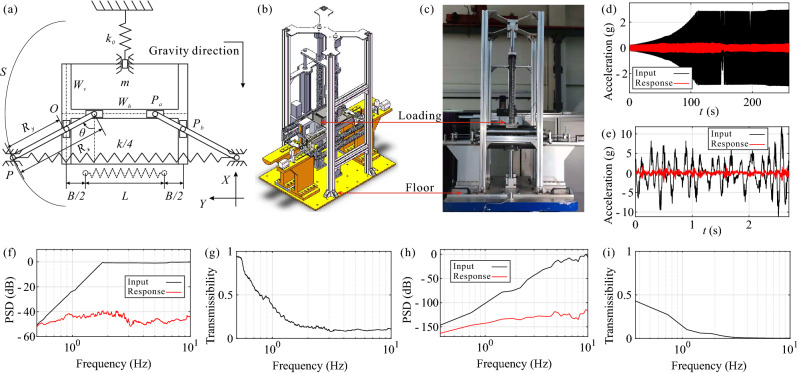
Experimental investigations (**a**) for the schematic of the experimental prototype, (**b**) for the CAD model of the experimental prototype, (**c**) for the panorama of the experimental prototype, (**d**) for the acceleration signal in time-domain of the frequency-sweep experiment, (**e**) for the acceleration signal in time-domain of the random vibration experiment, (**f**) for the PSD of the acceleration signal of the the frequency-sweep experiment, (**g**) for the acceleration transmissibility of the frequency-sweep experiment, (**h**) for the PSD of the acceleration signal of the the random vibration experiment, (**i**) for the acceleration transmissibility of the random vibration experiment.

An experimental prototype is built to demonstrate the unique dynamic characteristics of the ZS system based upon the schematic shown in Fig. 3a, which with the trajectory *S* of point *P* is an ellipse, which can be regarded as the coupling of a positive and a negative stiffness components^27,39,40^.

All the springs in the system are chosen as tension springs to avoid the stiffness deviation introduced by the buckling phenomenon of compression springs under compression. The transformation relationship of the horizontal spring stiffness from the original model and the redefinition of the geometric parameter *B*, caused by the application of tension springs instead of compression springs, are shown in the Fig. 3a.

**Figure 4:**
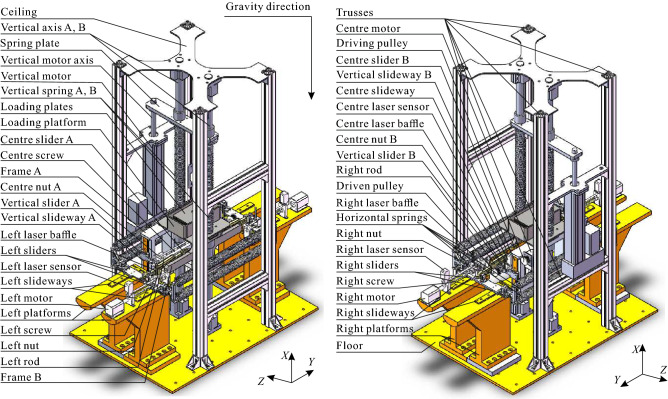
CAD model of the ZS system.

An experimental prototype was established based on the ZS system conditions shown in Fig. 4, in which the positive and negative directions of *X*-axis are defined as the directions of up and down respectively; the positive and negative directions of *Y*-axis are defined as the directions of right and left respectively; the positive and negative directions of *Z*-axis are defined as the directions of back and front respectively; and the following are the same.

The prototype shown in Fig. 4 is mainly composed of three parts, called the core-component, external-frame, and auxiliary-adjustment-system, respectively.

The core-component is composed of frame A, frame B, loading, a pair of connecting rods, a pair of vertical springs and two pairs of horizontal springs, which is used to verify the dynamic characteristics of the mechanical model shown in Fig. 3a. A pair of vertical slideways are respectively installed on the left and right sides of the frame A. The centre slideway is installed on the rear surface along the horizontal centre line of the frame B. The vertical slider A and B are respectively installed on the vertical slideway A and B, and the centre slider A and B are simultaneously installed on the centre slideway. The loading platform is mounted across the frame A and frame B, and appropriate loading plates are mounted inside the loading platform. Frame A and frame B are connected with vertical axis A and vertical axis B by linear bearing, respectively, to ensure the whole composed of frame A, B, and loading can only slide along the *X*-axis. The two vertical springs are respectively installed on the outside of the vertical axis A and B, and the upper and lower ends of the springs are separately fixed at the lower surface of the spring plate and the upper surface of the loading platform by clips. The horizontal springs connect the left sliders and the right sliders, which are separately installed on the left slideways and right slideways, by the hooks at both ends. The rear end of the spring plate is matched with the vertical motor axis, and the front end is matched with the vertical axis A and B by the linear bearings, respectively. The left (or right) end and the right (or left) end of the left (or right) rod are respectively connected with the left (or right) slider and the centre slider A (or B) by bearings. The two ends of the centre screw are separately fixed with the vertical slider A and B, and the left and right sides of the centre screw are respectively processed with left-hand and right-hand screw threads. The back ends of the centre nut A and B are separately matched with both sides of the centre screw by threads, and the front ends are respectively embedded in the middle grooves of the left and right rods by bearings, to make sure the front ends of the centre nut A and B can be locked in a certain position in each rod groove by bolts.

The external-frame is composed of ceiling, trusses, left and right platforms, and floor, which is used to carrying the core-components of the prototype and connecting vibration platform. The lower surface of the floor is fixed with the vibration platform, and the upper surface is fixed with the bottom of the vertical axis A and B by flanges, left and right platforms by bolts, and the bottom of the trusses. The left and right slideways are separately installed on the top surface of the left and right platforms. The lower surface of the ceiling is fixed with the top of the vertical axis A and B by flanges, and the top of the trusses.

**Figure 5:**
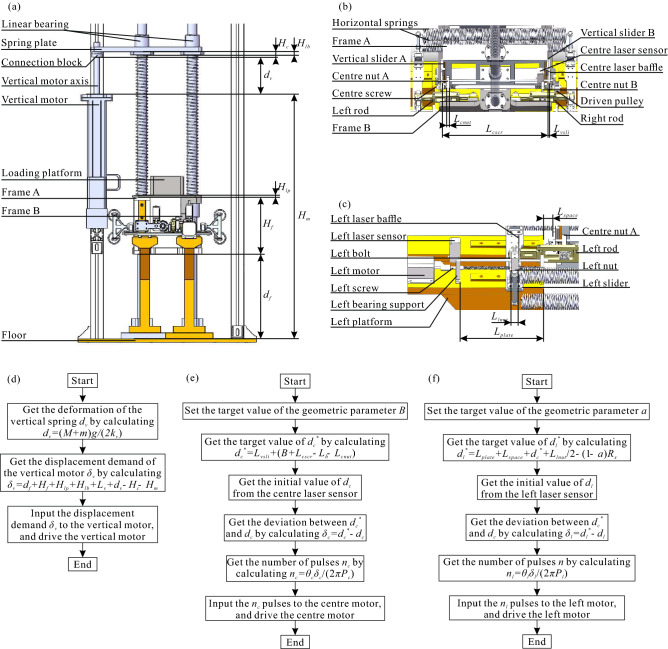
Details of the control systems of the prototype (**a**) for the partial enlarged of the vertical motor adjusting structure; (**b**) for the partial enlarged of the centre motor adjusting structure; (**c**) for the partial enlarged of the left motor adjusting structure; (**d**) for the logic block diagram of control strategy of the vertical motor adjusting structure; (**e**) for the logic block diagram of control strategy of the centre motor adjusting structure; and (**f**) for the logic block diagram of control strategy of the left motor adjusting structure.

The auxiliary-adjustment-system is composed of three groups of adjusting structures, which are used to adjust the geometric parameters of the system.

The detail of the vertical adjusting structure is shown in the right view of the experimental prototype, Fig. 5a. The vertical motor is fixed above a horizontal truss, which can drive the vertical motor axis to rotate and control the spring plate to move up and down along the *X*-axis direction, to adjust the vertical height assembly consisted of frame A, frame B, and loading. The logic block diagram of control strategy is shown in the Fig. 5d, in which $M\approx 27$ kg is the total mass of the frame A, frame B, loading platform, and other connecting structures installed on it, and the following are the same;

*m* is the total mass of the loading plates installed in the loading platform; $k_v=0.52$ N/mm is the stiffness of the single vertical spring; $d_f=265$ mm is the distance in *X*-axis between the upper surface of the floor and the lower surface of the frame A (or frame B) when both of the left and right rods are horizontal; $H_f=180$ mm is the height (‘height’ means the size in *x*-axis, and the following are the same) of the frame A (or frame B); $H_{lp}=5$ mm is the height of the base of the loading platform; $H_{lb}=10$ mm is the height of the base of the linear bearing installed in the spring plate; $L_v=290$ mm is the equilibrium length of each of the vertical spring; $H_c=17$ mm is the height of the connection block; and $H_m=773$ mm is the distance between the upper surface of the vertical motor and the upper surface of the floor.

The detail of the centre adjusting structure is shown in Fig. 5b. The centre motor is installed on a vertical truss, and its axis is fixed with the inner surface of the driving pulley. The inner surface of the driven pulley is fixed with the centre screw, and the outer surface is connected with the outer surface of the driving pulley through a belt. The centre laser sensor and the centre laser baffle are fixed on the vertical slider B and the centre nut B respectively. Then the centre screw can be driven by the centre motor to rotate, and the geometric parameter *B* shown in Fig. 3a can be adjusted indirectly based upon the distance signal between the vertical slider B and the centre nut B measured by the centre laser sensor. The logic block diagram of control strategy is shown in the Fig. 5e, in which $L_h=332.50$ mm expresses the equilibrium length of each of the horizontal spring; $L_{cscr}=387$ mm expresses the distance between the vertical slider A and B; $L_{vsli}=5$ mm and $L_{cnut}=10$ mm express the length in *Y*-axis of the vertical slider B and the centre nut B, respectively; $\theta _c=\dfrac{\pi }{100}$ rad expresses the step angle of the centre motor; and $P_c=2$ mm expresses the pitch of the centre screw.

The detail of the bilateral adjusting structure is shown in Fig. 5c, taking the left side as an example because of the symmetry of the prototype. The left motor is installed on one of the left platform near the front, and its axis is fixed with the left screw by a coupling. The left laser sensor and the left laser baffle are fixed on the left bearing support and the left nut respectively. The left slider and the left nut can be bounded by the left bolt. It is obviously that the displacement of the left rod relative to the centre nut A is the same as that of the left slider relative to the left bearing support, when both of the left and right rods are horizontal. In this case, the position of the left nut relative to the left rod $a=\dfrac{R_y}{R_x}$ can be adjusted indirectly by rotating the left screw through the left motor, based upon the distance signal measured by the left laser sensor. The logic block diagram of control strategy is shown in the Fig. 5f, in which $L_{plate}=189$ mm expresses the distance between the right surface of the left laser sensor and the right surface of the left platform in *Y*-axis; $L_{space}=21.50$ mm expresses the distance between the right surface of the left platform and the left surface of the vertical slider A in *Y*-axis; $L_{lnut}=16$ mm expresses the length of the left nut in *Y*-axis; $R_x=200$ mm expresses the centre distance of bearing at both ends of the left rod; $\theta _l=\dfrac{\pi }{100}$ rad expresses the step angle of the left motor; and $P_l=2$ mm expresses the pitch of the left screw.

The assembly process of the whole prototype can be described as following

1. Assemble the experiment prototype without installing the driven pulley, the belt, the centre laser sensor, the centre laser baffle, and any spring and loading plate, based upon the structure shown in Fig. 4;
2. Measure the mass *M* by weighing;
3. Install the vertical springs and loading plates;
4. Adjust the height of the spring plate by inputting mass parameters *M* and *m* into the control system of the vertical adjusting structure, to keep both the left and right rods horizontal;
5. Lock the installation position of the spring plate and turn off the vertical motor;
6. Install the driven pulley, the centre laser sensor, and centre laser baffle, after placing a supporting block with the height of $d_f$ between the frame A (or B) and the floor;
7. Connect the driving and the driven pulley by the belt, and ensure the belt is tightened by adjusting and locking the height of the centre motor;
8. Adjust the distance between centre nut A and B by inputting the target value of the geometric parameter *B* into the control system of the centre adjusting structure, then turn off the centre motor;
9. Unlock both the centre nuts A and B from the left and right rods, and bound the left and right nuts with the left and right sliders by installing the left and right bolts;
10. Adjust the relative position between the left (and right) rod and the centre nut A (and B), by inputting the target value of the geometric parameter *a* into the control system of the bilateral adjusting structure;
11. Lock the installation positions of the centre nut A and B relative to the left and right rods respectively, by tightening the bolts on the left and right rods, then turn off the left and right motors;
12. Disassemble left and right bolts, the belt, the driven pulley, the centre laser sensor, the centre laser baffle, and the supporting block;
13. Install the horizontal springs, acceleration sensors, and prepare to start the experiment.

The prototype with the adjustable loading 27 kg $\le M+m\le 37$ kg, the total stiffness of the vertical springs $k_0=1.04$ N/mm, the total stiffness of the horizontal springs $k/4=1$ N/mm, maximum stroke $l_0=R_x=200$ mm, minimum pre-stretch of horizontal springs $B=0$ mm, and the semi-minor axis $R_y=102$ mm. It is assembled on the top of a hydraulic vibration platform that provides external forces to the prototype at controlled amplitudes and frequencies. Two groups of acceleration sensors are mounted on the floor and the loading of the prototype to measure the input and the response signals of the vibration platform and the prototype, respectively, shown in Fig. 3b,c.

The vibration isolation capacity of the prototype is validated through a controlled frequency-sweep and a random spectrum input, where the input and the output signals are plotted in the black and red curves in Fig. 3d–f,h. The results of the frequency-sweep experiment are shown in Fig. 3d,f that the acceleration responses have been attenuated greatly with the maximum amplitude attenuation of $86.90\%$, and the vibration intensity is attenuated about 1.3 dB, 19.03 dB, and 43.90 dB at the frequencies of 0.5 Hz, 1 Hz, and 10 Hz, respectively. The acceleration transmissibility of the frequency-sweep experiment is shown in Fig. 3g, which implies a significant result that there is no amplification phenomenon caused by resonance in the frequency band higher than 0.5 Hz, which means that the initial vibration isolation frequency of this prototype is lower than 0.5 Hz (the lowest controllable output frequency of the vibration platform is 0.5 Hz). It can be noticed from the time-domain spectrum of the random vibration experiment, shown in Fig. 3e, that the peak value of the acceleration signal has been attenuated by about $80.0\%$ which demonstrated a high vibration isolation efficiency. On the other hand, it is observed from the frequency-domain spectrum, shown in Fig. 3h,i, that the vibration attenuation phenomenon is emerged markedly since about 0.37 Hz (calculated based on the sampling frequency of the acceleration sensors) obviously, which means that the initial vibration isolation frequency of the prototype is much lower than 0.37 Hz; and the vibration attenuation of the prototype is about 16.86 dB, 45.63 dB, and 112.37 dB at the frequencies of 0.37 Hz, 1 Hz, and 10 Hz, respectively, which provides the evident global isolation feature.

## Conclusions and outlooks

We have proposed a novel inverse construction method based upon the requirements of a supporting system, for design a passive system with arbitrary order QZS features to weaken the resonance. The proposed ZS system without affecting supporting that outright solve the problems introduced by resonance phenomena, which has plagued the engineering industry for nearly 300 years since 1750. Loads supported by this system have been isolated from environmental oscillations in the entire frequency-band, which expresses great significance in the field of ultra low-frequency vibration isolation. Apart from potential uses in vibration isolation, we have shown that a careful choice of parameter design allows the simulation of gravity-like force possible with arbitrary gravity or gravity-free environments. The reliability of the inverse method has been demonstrated theoretically and experimentally, which can even adapt the design for a more targeted application based on an arbitrary complex dynamic requirement. The unprecedented vibration isolation performance of this system opens new doors for engineering, and its distinctive geometric characteristics may lead to a new inspiration for the design of resonance-free metamaterial unit. Frictional forces between components will negatively impact performance, and implementations that seek to solve practical engineering problems may have to start there.

The experimental prototype with complex mechanical structure in this paper has been proposed to verify the dynamics characteristics and structural accuracy of the zero-stiffness system, which may introduce high-frequency vibration modes. Therefore, the prototype is being actively studied by the authors in structural optimization and improved design, to reduce the impact on the high-frequency vibration isolation performance caused by the complex structure, and further broaden the effective vibration isolation frequency band of the system.

## Methods

### Mathematical modeling of the designable archetype

Establish the coordinate system at the geometric centre of the block shown in Fig. 1 as its origin, and take the opposite and orthogonal direction of the gravity as the positive direction of the *X* and *Y* axis, respectively.

The displacement of the block relative to the initial state is assumed to be *X*, then the value of the resilience provided by the vertical spring can be obtained, written as

31 $$\begin{aligned} \left| \overrightarrow{F_v}\right| =k_0\left( X-B_0\right) , \end{aligned}$$

**Figure 6:**
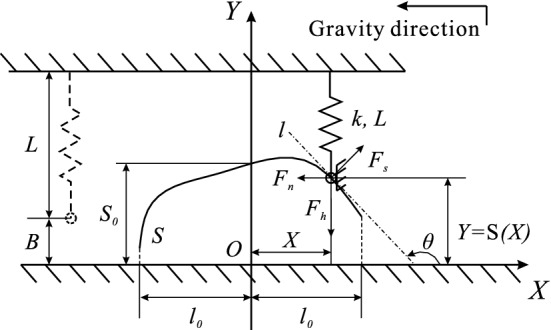
Simplified schematic diagram for the designable archetype.

The model can be simplified as the Fig. 6 shown, based upon the symmetry of the original model in *X* axis direction. The trajectory of the slideway *S* can be regarded as an infinitely differentiable function, $Y=S\left( X\right)$, with the definition domain $X\in \left( -l_0,l_0\right)$, where $l_0>0$, and the intercept of the curve on the *Y* axis is $S_0$.

The slider is subjected to three forces, $\overrightarrow{F_h}$, $\overrightarrow{F_s}$, and $\overrightarrow{F_n}$, provided by the horizontal spring, the slideway, and the basement, respectively, and satisfying the relation as following

32 $$\begin{aligned} \overrightarrow{F_n}=\overrightarrow{F_h}+\overrightarrow{F_s}. \end{aligned}$$

The force $\overrightarrow{F_h}$, $\overrightarrow{F_s}$, and $\overrightarrow{F_n}$ are perpendicular to the axis *X*, the curve *S*, and the axis *Y*, respectively, thus, the value of the elastic force $\overrightarrow{F_h}$ provided by the horizontal spring can be obtained as following

33 $$\begin{aligned} \left| \overrightarrow{F_h}\right| =k\left( S\left( X\right) -B\right) . \end{aligned}$$

The relationship between $\left| \overrightarrow{F_n}\right|$ and $\left| \overrightarrow{F_h}\right|$ can be written as the following

34 $$\begin{aligned} \frac{\left| \overrightarrow{F_n}\right| }{\left| \overrightarrow{F_h}\right| }=\tan \left( \pi -\theta \right) =-\tan \theta , \end{aligned}$$

based upon the relation of (32), where $\theta$ is the angle from the axis *X* to the line *l*, in which *l* is the tangent line of the curve *S* at the point of $\left( X,S\left( X\right) \right)$. The slope $k_l$ of the line *l* can be expressed by the curve equation as

35 $$\begin{aligned} k_l=\tan \theta =S'\left( X\right) . \end{aligned}$$

Substituting (33) and (35) into (34), the value of the force $\overrightarrow{F_n}$ is shown as

36 $$\begin{aligned} \left| \overrightarrow{F_n}\right| =-k\left( S\left( X\right) -B\right) S'\left( X\right) . \end{aligned}$$

The value of the resultant force $F\left( X\right)$ of the block provided by the vertical and the horizontal springs can be obtained, written as

37 $$\begin{aligned} F\left( X\right)&=-\left| \overrightarrow{F_n}\right| +\left| \overrightarrow{F_v}\right| +mg \\&=k\left( S\left( X\right) -B\right) S'\left( X\right) +k_0\left( X-B_0\right) +mg. \end{aligned}$$

Thus, the motion equation of the conservative system can be written as

38 $$\begin{aligned} m\frac{{\mathrm{d}}^2X}{{\mathrm{d}}t^2}+k\left( S\left( X\right) -B\right) S'\left( X\right) +k_0X+mg-k_0B_0=0. \end{aligned}$$

Then the equation (38) can be rewritten into the following form

39 $$\begin{aligned} \frac{{\mathrm{d}}^2x}{{\mathrm{d}}\tau ^2}+\kappa \left( \frac{1}{l_0}S\left( l_0x\right) -b\right) S'\left( l_0x\right) +x+\eta =0. \end{aligned}$$

An infinitely differentiable function $s\left( x\right)$ is defined as

40 $$\begin{aligned} s\left( x\right) =\frac{1}{l_0}S\left( l_0x\right) , \end{aligned}$$

and assume the value $s\left( 0\right) =s_0$. The following relation is acquired,

41 $$\begin{aligned} s'\left( x\right) =\frac{{\mathrm{d}}\left( \frac{1}{l_0}S\left( l_0x\right) \right) }{{\mathrm{d}}x}=\frac{1}{l_0}S'\left( l_0x\right) \frac{{\mathrm{d}}\left( l_0x\right) }{{\mathrm{d}}x}=S'\left( l_0x\right) , \end{aligned}$$

by differentiating both the sides of (40) simultaneously.

Then the dimensionless dynamic equation of (38) can be transformed, by substituting (41) and (40) into (39), written as

42 $$\begin{aligned} \ddot{x}+\kappa \left( s\left( x\right) -b\right) s'\left( x\right) +x+\eta =0. \end{aligned}$$

### Construction of QZS$^n$

The resultant force of system (1) written as following

43 $$\begin{aligned} f\left( x\right) =\kappa \left( s\left( x\right) -b\right) s'\left( x\right) +x+\eta , \end{aligned}$$

which can be expanded by Taylor formula at $x=0$ as

44 $$\begin{aligned} f\left( x\right) =\sum _{i=0}^\infty T_ix^i, \end{aligned}$$

where the coefficients $T_i$ ($i\in {\mathbb {N}}$) can be expressed as following

45 $$\begin{aligned} {\left\{ \begin{array}{ll} T_0=\kappa \left( s_0-b\right) s_1+\eta , \\ T_1=\kappa \left( s_1^2+s_0s_2-b s_2\right) +1, \\ T_i=\frac{\kappa }{i!}\left( \sum \limits _{j=0}^iC_i^js_{i-j+1}s_{j}-b s_{i+1}\right) ,i\ge 2. \end{array}\right. } \end{aligned}$$

Firstly, consider the first equation in (2), to analyse the property of the function $s\left( x\right)$ adapted to the system with QZS$^n$ characteristics. The establishment conditions of the first equation of (2) can be analysed by substituting (45) into it, and the relations between the function $s\left( x\right)$ and the dimensionless parameters can be obtained as (4). Obviously, the equation (4) defined a series $\left\{ s_n\right\}$ determined by parameters $\eta$, *b*, $\kappa$, and $s_0$ through a recursive relation.

It is easy to prove that the *q* order derivative of the function

46 $$\begin{aligned} {_ns}\left( x\right) =\sum \limits _{\varepsilon =1}^{\tfrac{n+3}{2}}\left( a_\varepsilon \cos \varepsilon x+b_\varepsilon \sin \varepsilon x\right) , \end{aligned}$$

satisfies the relation as following

47 $$\begin{aligned} \frac{d^q {_ns}\left( x\right) }{dx^q}\bigg |_{x=0}={_ns}_q= {\left\{ \begin{array}{ll} \sum \limits _{\varepsilon =1}^{\tfrac{n+3}{2}}\left( -\varepsilon ^2\right) ^{\tfrac{q}{2}}a_\varepsilon ,q=2i, \\ \sum \limits _{\varepsilon =1}^{\tfrac{n+3}{2}}\varepsilon \left( -\varepsilon ^2\right) ^{\tfrac{q-1}{2}}b_\varepsilon ,q=2i+1, \end{array}\right. } \end{aligned}$$

where $i\in {\mathbb {N}}$ and $q\le n+1$. Connect the formulas which have the same right subscripts in (47) and (4), respectively, then the equations can be obtained as following

48 $$\begin{aligned} {\left\{ \begin{array}{ll} {_ns}_0=\sum \limits _{\varepsilon =1}^{\tfrac{n+3}{2}}a_\varepsilon =s_0, \\ {_ns}_1=\sum \limits _{\varepsilon =1}^{\tfrac{n+3}{2}}\varepsilon b_\varepsilon =s_1, \\ \vdots \\ {_ns}_{2i}=\sum \limits _{\varepsilon =1}^{\tfrac{n+3}{2}}\left( -1\right) ^i\varepsilon ^{2i}a_\varepsilon =s_{2i}, \\ {_ns}_{2i+1}=\sum \limits _{\varepsilon =1}^{\tfrac{n+3}{2}}\left( -1\right) ^i\varepsilon ^{2i+1}b_\varepsilon =s_{2i+1}, \\ \vdots \\ {_ns}_{n+1}=\sum \limits _{\varepsilon =1}^{\tfrac{n+3}{2}}\left( -1\right) ^{\tfrac{n+1}{2}}\varepsilon ^{n+1}a_\varepsilon =s_{n+1}, \\ {_ns}_{n+2}=\sum \limits _{\varepsilon =1}^{\tfrac{n+3}{2}}\left( -1\right) ^{\tfrac{n+1}{2}}\varepsilon ^{n+2}b_\varepsilon =s_{n+2}. \\ \end{array}\right. } \end{aligned}$$

Rewriting the Eq. (48) into the following form

49 $$\begin{aligned} {\textbf{V}}\cdot {\textbf{C}}= \begin{bmatrix} {\mathbf {V}}_{\mathbf{A}} &{}\quad {\textbf{0}} \\ {\textbf{0}} &{}\quad {\mathbf {V}}_{\mathbf{B}} \end{bmatrix} \begin{bmatrix} {\textbf{A}} \\ {\textbf{B}} \end{bmatrix}= \begin{bmatrix} {\mathbf {S}}_{\mathbf{A} }\\ {\mathbf {S}}_{\mathbf{B}} \end{bmatrix} ={\textbf{S}}. \end{aligned}$$

in which both the vectors ${\textbf{A}}$ and ${\textbf{B}}$ with $\dfrac{n+3}{2}$ rows satisfy

50 $$\begin{aligned} {\textbf{A}}= \begin{bmatrix} a_1&\quad a_2&\quad \cdots&\quad a_{\tfrac{n+3}{2}} \end{bmatrix}^{\mathrm{T}} \end{aligned}$$

and

51 $$\begin{aligned} {\textbf{B}}= \begin{bmatrix} b_1&\quad b_2&\quad \cdots&\quad b_{\tfrac{n+3}{2}} \end{bmatrix}^{\mathrm{T}}, \end{aligned}$$

respectively; both the vectors ${\mathbf {S}}_{\mathbf{A}}$ and ${\mathbf {S}}_{\mathbf{B}}$ with $\dfrac{n+3}{2}$ rows satisfy

52 $$\begin{aligned} {\mathbf {S}}_{\mathbf{A}}= \begin{bmatrix} s_0&\quad s_2&\quad \cdots&\quad s_{n+1} \end{bmatrix}^{\mathrm{T}} \end{aligned}$$

and

53 $$\begin{aligned} {\mathbf {S}}_{\mathbf{B}}= \begin{bmatrix} s_1&\quad s_3&\quad \cdots&\quad s_{n+2} \end{bmatrix}^{\mathrm{T}}, \end{aligned}$$

respectively; and both the matrices ${\mathbf {V}}_{\mathbf{A}}$ and ${\mathbf {V}}_{\mathbf{B}}$ with $\dfrac{n+3}{2}$ rows and columns, satisfy

54 $$\begin{aligned} {\mathbf {V}}_{\mathbf{A}}={\begin{bmatrix} 1 &{}\quad 1 &{}\quad \cdots &{}\quad 1 \\ -1 &{}\quad -2^2 &{}\quad \cdots &{}\quad -\left( \tfrac{n+3}{2}\right) ^2 \\ \vdots &{}\quad \vdots &{}\quad \ddots &{}\quad \vdots \\ \left( -1\right) ^{\tfrac{n+1}{2}} &{}\quad \left( -1\right) ^{\tfrac{n+1}{2}}2^{n+1} &{}\quad \cdots &{}\quad \left( -1\right) ^{\tfrac{n+1}{2}}\left( \tfrac{n+3}{2}\right) ^{n+1} \end{bmatrix}} \end{aligned}$$

and

55 $$\begin{aligned} {\mathbf {V}}_{\mathbf{B}}={\begin{bmatrix} 1 &{}\quad 2 &{}\quad \cdots &{}\quad \tfrac{n+3}{2} \\ -1 &{}\quad -2^3 &{}\quad \cdots &{}\quad -\left( \tfrac{n+3}{2}\right) ^3 \\ \vdots &{}\quad \vdots &{}\quad \ddots &{}\quad \vdots \\ \left( -1\right) ^{\tfrac{n+1}{2}} &{}\quad \left( -1\right) ^{\tfrac{n+1}{2}}2^{n+2} &{}\quad \cdots &{}\quad \left( -1\right) ^{\tfrac{n+1}{2}}\left( \tfrac{n+3}{2}\right) ^{n+2} \end{bmatrix}}, \end{aligned}$$

respectively. Apparently, any element of ${\mathbf {V}}_{\mathbf{A}}$ and ${\mathbf {V}}_{\mathbf{B}}$ located in the *i*th row and the *j*th column of the matrix can be expressed as $\left( -j^2\right) ^{i-1}$ and $j\left( -j^2\right) ^{i-1}$, respectively, which means that ${\mathbf {V}}_{\mathbf{A}}$ and ${\mathbf {V}}_{\mathbf{B}}$ are expressed as a kind of Vandermonde matrix and a part of a kind of Vandermonde matrix, respectively. Thus, it is easy to prove that the determinant of the matrix ${\textbf{V}}$ can be acquired as

56 $$\begin{aligned} \left| {\textbf{V}}\right| =\left| {\mathbf {V}}_{\mathbf{A}}\right| \left| {\mathbf {V}}_{\mathbf{B}}\right| , \end{aligned}$$

where

57 $$\begin{aligned} \left| {\mathbf {V}}_{\mathbf{A}}\right| =\prod \limits _{1\le j<i\le \tfrac{n+3}{2}}\left( j^2-i^2\right) \end{aligned}$$

and

58 $$\begin{aligned} \left| {\mathbf {V}}_{\mathbf{B}}\right| =\tfrac{n+3}{2}!\prod \limits _{1\le j<i\le \tfrac{n+3}{2}}\left( j^2-i^2\right) . \end{aligned}$$

Thus, we can draw the conclusion that the inverse of the matrix ${\textbf{V}}$ always exists, and the vector ${\textbf{C}}$, which consists of undetermined coefficient $\left\{ a_\varepsilon \right\}$ and $\left\{ b_\varepsilon \right\}$, always has a solution written as

59 $$\begin{aligned} {\textbf{C}}={\textbf{V}}^{-1}\cdot {\textbf{S}}. \end{aligned}$$

Secondly, the analysis of the second equation of (2) can be transformed into proof of the following proposition.

#### Proposition 2

*On the premise that the trajectory function of a system satisfies the definition of formula* (46), *for any function* $_ns\left( x\right)$ *with* $n+3$ *terms, if it satisfies* $T_i=0, i\le n+1$, *it must satisfy* $T_{n+2}\ne 0$.

#### Proof

Assuming there is *n* to make $T_{n+2}=0$, under the condition of the function $_ns\left( x\right)$ with $n+3$ terms which satisfied $T_i=0, i\le n+1$. There must be at least one vector ${\mathbf {A}}_{\mathbf{0}}$ with $\frac{n+3}{2}$ rows as the solution to hold the equation as following

60 $$\begin{aligned} {^+}{\mathbf{V}}_{\mathbf{A}}\cdot {\mathbf {A}}_{\mathbf{0}}={^+}{\mathbf{S}}_{\mathbf{A}}, \end{aligned}$$

where the vector $$ {^+}{\mathbf{S}}_{\mathbf{A}}= \begin{bmatrix} {\mathbf {S}}_{\mathbf{A}}^{\mathrm{T}}&s_{n+3} \end{bmatrix}^{\mathrm{T}} $$ with $\frac{n+5}{2}$ rows, and the matrix $$ {^+}{\mathbf{V}}_{\mathbf{A}}= \begin{bmatrix} \mathbf {V}_{\mathbf{A}}^{\mathrm{T}}&{\mathbf {V}}_{\mathbf{A}}^{*} \end{bmatrix}^{\mathrm{T}}$$ with $\frac{n+5}{2}$ rows and $\frac{n+3}{2}$ columns, in which $${\mathbf {V}}_{\mathbf{A}}^{*}=\left( -1\right) ^{\tfrac{n+3}{2}} \begin{bmatrix} 1&2^{n+3}&\cdots&\left( \frac{n+3}{2}\right) ^{n+3} \end{bmatrix}^{\mathrm{T}}$$. Hence, the solution ${\mathbf {A}}_{\mathbf{0}}$ of (60) must simultaneously hold both the formulas

61 $$\begin{aligned} \mathbf {^+_1V_A}\cdot \mathbf {A_0}=\mathbf {^+_1S_A} \end{aligned}$$

and

62 $$\begin{aligned}  {^+}_2{\mathbf{V}}_{\mathbf{A}}\cdot {\mathbf {A}}_{\mathbf{0}}= {^+}_2{\mathbf{S}}_{\mathbf{A}}, \end{aligned}$$

where the matrix ${^+}_i{\mathbf{V}}_{\mathbf{A}}$ represents a matrix with $\frac{n+3}{2}$ rows and columns, after the *i*th row is deleted from the matrix ${^+{\mathbf{V}}_{\mathbf{A}}}$. Because matrix $\mathbf {^+V_A}$ belongs to a kind of Vandermonde matrix, the inverse matrices of $\mathbf {^+_1V_A}$ and $\mathbf {^+_2V_A}$ are both exist. Therefore, the equation as following

63 $$\begin{aligned} \mathbf {^+_1V_A^{-1}}\cdot \mathbf {^+_1S_A}=\mathbf {^+_2V_A^{-1}}\cdot \mathbf {^+_2S_A} \end{aligned}$$

must holds. Then the following two formulas

64 $$\begin{aligned} \mathbf {_1V_A}\cdot {\textbf{A}}=s_0 \end{aligned}$$

and

65 $$\begin{aligned} \mathbf {_2V_A}\cdot {\textbf{A}}=s_2 \end{aligned}$$

must be equivalent, in which the vector $\mathbf {_iV_A}$ express the line *i* in matrix $\mathbf {V_A}$.

According to the features of the parameters $s_0$ and $s_2$ defined in formulas (4), and the vectors $\mathbf {_1V_A}$ and $\mathbf {_2V_A}$ defined in (54), it is evident that equations (64) and (65) are not equivalent. Consequently, the Proposition 2 is proved for the hypothesis is not valid. $\square$

Therefore, the QZS$^n$ system can always be constructed by introduce a curve function conforming to the form of (46), with the undetermined coefficients determined by (7).

### Inverse construction method

Represent $s\left( x\right)$ and $s'\left( x\right)$ by *s* and $\dfrac{{\mathrm{d}}s}{{\mathrm{d}}x}$, respectively, and separate the variables *s* and *x*, to rewrite the first equation in (11) as the following

66 $$\begin{aligned} \kappa \left( s-b\right) {\mathrm{d}}s=\left( f\left( x\right) -\eta -x\right) {\mathrm{d}}x. \end{aligned}$$

Performing indefinite integrals on both sides of the equation (66) simultaneously, there is

67 $$\begin{aligned} \kappa \left( s-b\right) ^2=2\int \left( f\left( x\right) -\eta \right) {\mathrm{d}}x-x^2+C, \end{aligned}$$

where *C* is an arbitrary constant. Assuming the function

68 $$\begin{aligned} G\left( x\right) =\int \left( f\left( x\right) -\eta \right) {\mathrm{d}}x+C \end{aligned}$$

is the integral primitive function of $f\left( x\right) -\eta$, then a relation can be achieved as following,

69 $$\begin{aligned} &\int \left( f\left( x\right) -\eta \right) {\mathrm{d}}x+C =G\left( x\right) -G\left( 0\right) +G\left( 0\right) \\&\quad =\int _0^x\left( f\left( t\right) -\eta \right) {\mathrm{d}}t+G\left( 0\right) +C \\&\quad =\int _0^x\left( f\left( t\right) -\eta \right) {\mathrm{d}}t+C. \end{aligned}$$

Substituting (69) and the second equation of (11) into (67), the arbitrary constant *C* can be obtained as following

70 $$\begin{aligned} C=\kappa \left( s_0-b\right) ^2. \end{aligned}$$

Thus, the solution of the initial value problem of the differential equations (11) can be written as following,

71 $$\begin{aligned} \kappa \left( \left( s\left( x\right) -b\right) ^2-\left( s_0-b\right) ^2\right) =2\int _0^x\left( f\left( t\right) -\eta \right) {\mathrm{d}}t-x^2. \end{aligned}$$

### Transition sets

The transition sets of the system (25) can be analysed by consider the following bifurcation conditions of equilibrium at $x_0$,

72 $$\begin{aligned} {\left\{ \begin{array}{ll} F\left( x_0\right) =0, \\ F'\left( x\right) \big |_{x=x_0}=0, \end{array}\right. } \end{aligned}$$

in which

73 $$\begin{aligned} F\left( x\right) =\left( 1-\alpha +\frac{\beta }{\sqrt{1-x^2}}\right) x \end{aligned}$$

and $F'\left( x\right)$ represent the resilience function and its derivative function, respectively, and the following are the same. Apparently, the equilibrium is non-hyperbolic and bifurcate, if

74 $$\begin{aligned} \alpha -\beta -1=0; \end{aligned}$$

moreover, the equilibrium is a saddle point when

75 $$\begin{aligned} \alpha -\beta -1<0, \end{aligned}$$

and otherwise it is a centre point.

Similarly, consider the following bifurcation conditions of equilibriums at $x_\pm$,

76 $$\begin{aligned} {\left\{ \begin{array}{ll} F\left( x_\pm \right) =0, \\ F'\left( x\right) \big |_{x=x_\pm }=0, \end{array}\right. } \end{aligned}$$

which can be reduced into the following forms

77 $$\begin{aligned} \alpha -\beta -1=0,0<\frac{\beta }{\alpha -1}\le 1. \end{aligned}$$

Ulteriorly, we can notice that the number of equilibrium can be changed by whether the existence condition of the solution,

78 $$\begin{aligned} 0<\frac{\beta }{\alpha -1}\le 1, \end{aligned}$$

holds. The solutions set of inequality (78) can be expressed as I and III in Fig. 2b. Notably, the system (25) will degenerate into a linear system, express as

79 $$\begin{aligned} \ddot{x}+\left( 1-\alpha \right) x=0, \end{aligned}$$

when the parameters’ combination $\left( \alpha ,\beta \right)$ falls into one of the boundaries, $\beta =0$, of the regions I and III. Therefore, a new bifurcation set can be acquired as

80 $$\begin{aligned} \beta =0, \end{aligned}$$

caused by the linear degenerate of a nonlinear system. Obviously, the equilibriums $x_\pm$ are saddle points when

81 $$\begin{aligned} {\left\{ \begin{array}{ll} \beta<0, \\ \alpha -\beta -1<0, \end{array}\right. } \end{aligned}$$

and they are centre points when

82 $$\begin{aligned} {\left\{ \begin{array}{ll} \beta>0, \\ \alpha -\beta -1>0. \end{array}\right. } \end{aligned}$$

Hence, the transition sets consisted by bifurcation values of parameters can be acquired in the parameter space $\left( \alpha ,\beta \right)$, as following

83 $$ \begin{aligned}&{\mathscr {B}}_1=\left\{ \left( \alpha ,\beta \right) |\alpha -\beta =1,\alpha>0,\beta<0\right\} , \\&{\mathscr {B}}_2=\left\{ \left( \alpha ,\beta \right) |\alpha -\beta =1,\beta>0\right\} , \\&{\mathscr {B}}_3=\left\{ \left( \alpha ,\beta \right) |\beta =0,0<\alpha <1\right\} , \\&{\mathscr {B}}_4=\left\{ \left( \alpha ,\beta \right) |\beta =0,\alpha >1\right\} ,\\&C=\left\{ \left( \alpha ,\beta \right) |\beta =0,\alpha =1\right\} . \end{aligned}$$

### Transmissibility

Consider the dimensionless non-conservative forced system with linear damping, which can be written as the following

84 $$\begin{aligned} \ddot{x}+2\zeta {\dot{x}}+\left( 1-\alpha +\frac{\beta }{\sqrt{1-x^2}}\right) x=f\cos \omega \tau , \end{aligned}$$

in which $\zeta$ is the damping ratio of the system. Assuming the velocity and displacement solutions are periodic, which can be indicated as following

85 $$\begin{aligned} {\left\{ \begin{array}{ll} x=\rho \cos \left( \omega \tau +\phi \right) , \\ v=-\omega \rho \sin \left( \omega \tau +\phi \right) , \end{array}\right. } \end{aligned}$$

which expressed by two variables of the amplitude $\rho$ and phase $\phi$, that can be assumed to be slowly varying functions of dimensionless time $\tau$. Thus, the first and second derivatives of *x* can be obtained, based on (85) as following

86 $$\begin{aligned} {\left\{ \begin{array}{ll} {\dot{x}}=\frac{{\mathrm{d}}x}{{\mathrm{d}}\tau }={\dot{\rho }}\cos \left( \omega \tau +\phi \right) -\rho \left( \omega +{\dot{\phi }}\right) \sin \left( \omega \tau +\phi \right) , \\ \ddot{x}=\frac{{\mathrm{d}}v}{{\mathrm{d}}\tau }=-\omega \left( {\dot{\rho }}\sin \left( \omega \tau +\phi \right) +\rho \left( \omega +{\dot{\phi }}\right) \cos \left( \omega \tau +\phi \right) \right) . \end{array}\right. } \end{aligned}$$

Substitute (86) and the first equation of (85) into the system (84), which can be rewritten (84) as following

87 $$\begin{aligned} -\omega \left( {\dot{\rho }}\sin \psi +\rho \left( \omega +{\dot{\phi }}\right) \cos \psi \right) +2\zeta \left( {\dot{\rho }}\cos \psi -\rho \left( \omega +{\dot{\phi }}\right) \sin \psi \right) +\left( 1-\alpha \right) \rho \cos \psi +\dfrac{\beta \rho \cos \psi }{\sqrt{1-\left( \rho \cos \psi \right) ^2}}=f\cos \left( \psi -\phi \right) , \end{aligned}$$

in which $\psi =\omega \tau +\phi$. Perform a Fourier expansion for the unjustifiable term

88 $$\begin{aligned} \dfrac{\beta \rho \cos \psi }{\sqrt{1-\left( \rho \cos \psi \right) ^2}}=F_0+\sum _{n=1}^\infty \left( F_{a_n}\cos n\psi +F_{b_n}\sin n\psi \right) , \end{aligned}$$

where

89 $$\begin{aligned} F_0=\frac{1}{2\pi }\int _0^{2\pi }\dfrac{\beta \rho \cos \psi }{\sqrt{1-\left( \rho \cos \psi \right) ^2}}{\mathrm{d}}\psi , \end{aligned}$$

90 $$\begin{aligned} F_{a_n}=\frac{1}{\pi }\int _0^{2\pi }\dfrac{\beta \rho \cos \psi }{\sqrt{1-\left( \rho \cos \psi \right) ^2}}\cos n\psi {\mathrm{d}}\psi , \end{aligned}$$

and

91 $$\begin{aligned} F_{b_n}=\frac{1}{\pi }\int _0^{2\pi }\dfrac{\beta \rho \cos \psi }{\sqrt{1-\left( \rho \cos \psi \right) ^2}}\sin n\psi {\mathrm{d}}\psi . \end{aligned}$$

It is easy to prove that $${\left\{ \begin{array}{ll} F_0\equiv 0 \\ F_{b_n}\equiv 0 \end{array}\right. }$$ , by considering the integral formulas (89) and (91). The approximate system can be obtained by ignoring the influence of higher harmonic terms of the equation (88), written as

92 $$\begin{aligned} -\omega \left( {\dot{\rho }}\sin \psi +\rho \left( \omega +{\dot{\phi }}\right) \cos \psi \right) +2\zeta \left( {\dot{\rho }}\cos \psi -\rho \left( \omega +{\dot{\phi }}\right) \sin \psi \right) +\left( 1-\alpha +\frac{\beta }{\pi }Z\left( \rho \right) \right) \rho \cos \psi =f\cos \left( \psi -\phi \right) , \end{aligned}$$

in which

93 $$\begin{aligned} Z\left( \rho \right) =\int _0^{2\pi }\dfrac{\cos ^2\psi }{\sqrt{1-\rho ^2\cos ^2\psi }}{\mathrm{d}}\psi =\frac{4}{\rho ^2}\left( \frac{1}{\sqrt{1-\rho ^2}}{\mathrm{EllipticK}}\left( \frac{\rho ^2}{-1+\rho ^2}\right) -\sqrt{1-\rho ^2}{\mathrm{EllipticE}}\left( \frac{\rho ^2}{-1+\rho ^2}\right) \right) , \end{aligned}$$

and ${\mathrm{EllipticK}}\left( k\right)$, ${\mathrm {EllipticE}}\left( k\right)$ denote the complete elliptic integral of the first and second kinds with the elliptic module *k*, respectively. Making the coefficients of the same harmonic terms in system (92) equal, the formulas describing the derivative of amplitude and phase can be obtained as following

94 $$\begin{aligned} {\left\{ \begin{array}{ll} {\dot{\rho }}=\frac{2\pi \zeta \left( \left( \alpha -1\right) \rho +f\cos \phi \right) -\pi \omega f\sin \phi -2\zeta \beta \rho Z\left( \rho \right) }{\pi \left( 4\zeta ^2+\omega ^2\right) }, \\ {\dot{\phi }}=\frac{-\pi \left( \omega \rho \left( \alpha -1+4\zeta ^2+\omega ^2\right) +\omega f\cos \phi +2\zeta f\sin \phi \right) +\beta \omega \rho Z\left( \rho \right) }{\pi \rho \left( 4\zeta ^2+\omega ^2\right) }. \end{array}\right. } \end{aligned}$$

Let $${\left\{ \begin{array}{ll} {\dot{\rho }}=0 \\ {\dot{\phi }}=0 \end{array}\right. }$$ that derive singular point $\left( \rho _s,\phi _s\right)$ that corresponds to the periodic solution of steady state of system (84), which satisfies

95 $$\begin{aligned} {\left\{ \begin{array}{ll} f\cos \phi =\rho \left( 1-\alpha -\omega ^2+\frac{\beta }{\pi }Z\left( \rho \right) \right) , \\ f\sin \phi =-2\zeta \omega \rho , \end{array}\right. } \end{aligned}$$

from which the amplitude-frequency relationship for the approximate system (92) is obtained by elimination of phase angle based upon (95), as following

96 $$\begin{aligned} \left( 1-\alpha -\omega ^2+\frac{\beta }{\pi }Z\left( \rho \right) \right) ^2+\left( 2\zeta \omega \right) ^2=\frac{f^2}{\rho ^2}. \end{aligned}$$

Evidently, the equation of force transmissibility relationship can be acquired as following

97 $$\begin{aligned} T^2=\frac{\left( 1-\alpha +\frac{\beta }{\pi }Z\left( \dfrac{Tf}{\omega ^2}\right) \right) ^2+\left( 2\zeta \omega \right) ^2}{\left( 1-\alpha +\frac{\beta }{\pi }Z\left( \dfrac{Tf}{\omega ^2}\right) -\omega ^2\right) ^2+\left( 2\zeta \omega \right) ^2}. \end{aligned}$$

### Demonstration for the elliptical trajectory in prototype

Establishing the coordinate system *XOY* by taking *O* as the origin, the vertical upward direction as the positive direction of *X* axis, and the horizontal left direction as the positive direction of *Y* axis. Defining the angle from the negative direction of *X* axis to the left rod as $\theta$. Then the trajectory *S* of point *P* relative to the origin *O* can be expressed as

98 $$\begin{aligned} {\left\{ \begin{array}{ll} x=R_x\cos \theta , \\ y=R_y\sin \theta . \end{array}\right. } \end{aligned}$$

Substituting the identity relation $\sin ^2\theta +\cos ^2\theta \equiv 1$ into (98), and simplifying. Then the trajectory equation of point *P* can be obtained as following

99 $$\begin{aligned} \dfrac{x^2}{R_x^2}+\dfrac{y^2}{R_y^2}=1. \end{aligned}$$

Thus, the trajectory *S* of point *P* is an ellipse.

### Conditions of experiments

**Figure 7:**
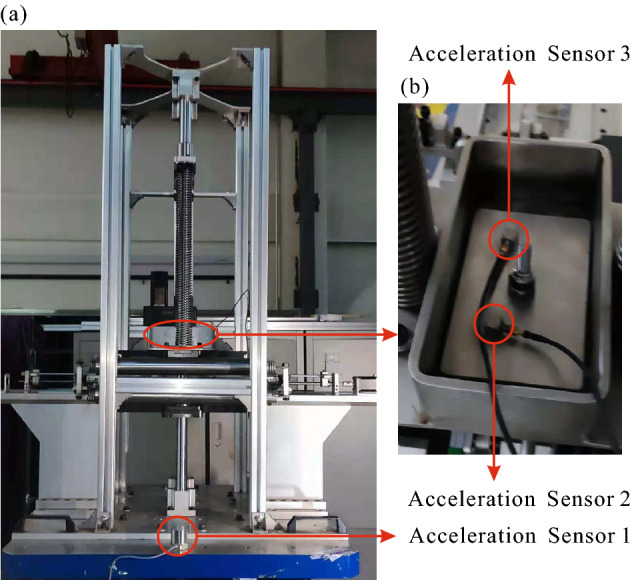
Experimental prototype and sensor layout scheme (**a**) for the panorama of the assembled prototype and the sensor layout scheme, (**b**) for the partial enlarged view of the loading platform.

The experimental prototype and sensor layout scheme are shown in Fig. 7, in which (a) for the panorama of the prototype and (b) for the partial enlarged view of the loading platform. The acceleration sensor 1 mounted on the vibration platform to measure the acceleration signal of the vibration platform in the *X*-axis and to control the vibration platform. The acceleration sensors 2 and 3 are mounted on the top of the loading plate to measure the acceleration signal of the loading response by averaging.

**Figure 8:**
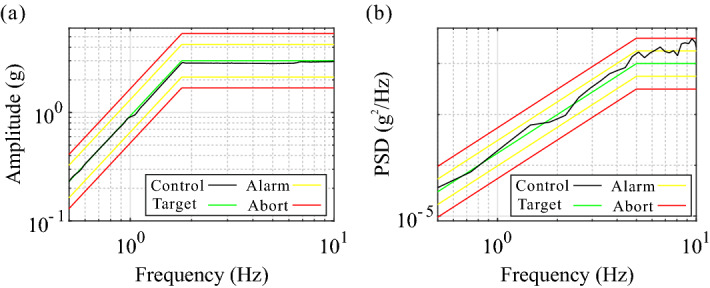
Target spectrum of the control signal (**a**) for the frequency-sweep experiment, (**b**) for the random vibration experiment.

The target spectrum of the control signal for frequency-sweep experiment is set according to the following rules:

1. The vibration platform with a constant displacement amplitude equals to 230 mm, when the acceleration amplitude is not higher than 3 g in low-frequency band;
2. The vibration platform with a constant acceleration amplitude equals to 3 g, after the acceleration amplitude reaches 3 g for the first time, since the frequency about 1.8 Hz; which is shown as the green curve in Fig. 8a, and the black, yellow, and red curves represents the control signal of the acceleration sensor 1, the alarm threshold, and the abort threshold, respectively.

The target spectrum of the control signal for random vibration experiment is set according to the following rules:

1. The PSD of the control signal increases linearly from $3\times 10^{-5}$ g$^2$/Hz to $1\times 10^{-2}$ g$^2$/Hz in the frequency band of 0.5–5 Hz;
2. The PSD of the control signal remains $1\times 10^{-2}$ g$^2$/Hz in the frequency band of 5–10 Hz; which is shown as the green curve in Fig. 8b, and the black, yellow, and red curves represents the control signal of the acceleration sensor 1, the alarm threshold, and the abort threshold, respectively.

## Supplementary Information

Supplementary Video 1.

Supplementary Video 2.

Supplementary Legends.

## Data Availability

All data that led us to understand the results presented here are available with the paper or from the corresponding author upon reasonable request. Source data are provided with this paper.
