# Supplementary material for: Road to entire insulation for resonances from a forced mechanical system
Source: Sci Rep. 2022 Dec 7;12:21167. doi: 10.1038/s41598-022-25691-4 (PMC9729684; doi:10.1038/s41598-022-25691-4)
Supplement: Supplementary file 3 — Supplementary Legends. [file 41598_2022_25691_MOESM3_ESM.pdf]

Legends for the video files

1. The video named “dp.mp4” is the constant frequency vibration experiment video with the frequency of 1Hz;

2. The video named “sj.mp4” is the random vibration experiment video with frequency range of 0.37Hz to 10Hz.
